# Glial S100A6 Degrades β-amyloid Aggregation through Targeting Competition with Zinc Ions

**DOI:** 10.14336/AD.2018.0912

**Published:** 2019-08-01

**Authors:** Zhi-Ying Tian, Chun-Yan Wang, Tao Wang, Yan-Chun Li, Zhan-You Wang

**Affiliations:** ^1^Institute of Health Sciences, Key Laboratory of Medical Cell Biology of Ministry of Education, China Medical University, Shenyang 110122, China; ^2^Department of Medicine, The University of Chicago, Chicago, IL 60637, USA

**Keywords:** S100A6, β-amyloid protein, astrocyte, zinc, Alzheimer’s disease

## Abstract

Evidence has been accumulating that zinc ions can trigger β-amyloid (Aβ) deposition and senile plaque formation in the brain, a pathological hallmark of Alzheimer’s disease (AD). Chelating zinc inhibits Aβ aggregation and may hold promise as a therapeutic strategy for AD. S100A6 is an acidic Ca^2+^/Zn^2+^-binding protein found only in a small number of astrocytes in the normal brain. However, in the AD brain, S100A6 is highly expressed in astrocytes around Aβ plaques. The role of the astrocytic S100A6 upregulation in AD is unknown. In the present study, we examined the effects of S100A6 on Aβ plaques and intracellular zinc levels in a mouse model of AD. Chronic exposure to zinc increased Aβ deposition and S100A6 expression, both reversible by the zinc chelator clioquinol, in the brains of amyloid precursor protein/presenilin 1 (APP/PS1) transgenic mice. To examine whether exogenous S100A6 could induce Aβ plaque disaggregation through competition for zinc in vitro, we incubated APP/PS1 mouse brain sections with recombinant human S100A6 protein or co-incubated them with human S100A6-expressing cells. Both treatments efficiently reduced the Aβ plaque burden in situ. In addition, treatment with exogenous S100A6 protected cultured COS-7 cells against zinc toxicity. Our results show for the first time that increased S100A6 levels correlate with both Aβ disaggregation and decrease of Aβ plaque-associated zinc contents in brain sections with AD-like pathology. Astrocytic S100A6 in AD may protect from Aβ deposition through zinc sequestration.

Alzheimer’s disease (AD) is a heterogeneous, progressive neurodegenerative disorder that represents the most common cause of dementia in the elderly [1, 2]. A primary theory for the cause of AD is the overload of extracellular amyloid-β (Aβ) peptides in the brain [3, 4]. Aβ aggregation into cytotoxic amyloid fibers or protofibrils may be an important step in AD progression [5, 6]. The dyshomeostasis of some metals, such as copper, zinc, and iron, is commonly found in the AD brain [7-9]. Several studies have demonstrated that the interaction of Aβ with these metals can increase its neurotoxic effects as a consequence of marked biophysical alterations of the peptide [10, 11]. Aβ contains zinc-binding sites. Increasing the concentration of zinc ions can trigger Aβ deposition [12-16], facilitating the formation of senile plaques in the brain. *In vitro* studies using synthetic monomeric Aβ have shown that zinc may accelerate Aβ aggregation into oligomeric species, which may eventually form fibrils [17]. Based on the potential role of zinc in the deposition of Aβ in the AD brain, there has been considerable interest in the use of metal chelators to decrease amyloid neuropathology [18-21]. Metal-chelating agents, such as clioquinol (CQ) and deferoxamine, have been shown to inhibit the formation of amyloid plaques in the brains of amyloid precursor protein/presenilin 1 (APP/PS1) transgenic mice [22, 23] and *in vitro* [24]. Therefore, it has been suggested that the modulation of intracranial zinc availability might be a potential therapeutic strategy in AD, and the disruption of zinc homeostasis in the brain may be an early and necessary step in the initiation of Aβ accumulation.

S100A6 is a small (10 kDa) EF-hand acidic Ca^2+^/Zn^2+^-binding protein that belongs to the large S100 protein family [25, 26]. In the central nervous system, S100A6 is mainly expressed in the amygdala and entorhinal cortex neurons while it is also found in some astrocytes [27]. Interestingly, S100A6 upregulation in astrocytes has been observed in patients suffering from amyotrophic lateral sclerosis (ALS) [28], and around amyloid plaque deposits in AD patients and transgenic AD model animals [29]. These findings suggest that S100A6 may be involved in the pathogeneses of both AD and ALS. However, the effects of the astrocytic S100A6 upregulation in AD and ALS are unclear. S100A6 has been reported to have two zinc-binding sites and a higher affinity for zinc than calcium ions [30]. Zn^2+^ binding induces conformational changes in the S100A6 molecule [31-33]. Boom and colleagues observed that S100A6 clusters were correlated to the density of Aβ-associated senile plaques [29], whereas the effect of S100A6 overexpression in zinc levels is not clear yet. Given the high zinc-binding capacity of S100A6, we proposed that the elevated expression of S100A6 around amyloid plaques in patients with AD might play an important role in inhibiting Aβ aggregation and plaque formation through competition for zinc. We hypothesized that the competing pattern may protect neurons from the neurotoxic damage of zinc-triggered Aβ deposition. In the present study, we aimed to characterize the effects of S100A6 on zinc levels and Aβ aggregation in the brains of APP/PS1 transgenic mice. The ability of S100A6 to promote plaque disaggregation and protect against zinc-induced toxicity was evaluated *in vitro.*

## MATERIALS AND METHODS

### Animals and treatments

Male APP/PS1 double-transgenic mice (Jackson Laboratory, USA) were used in the present study. The mice were kept in cages in a controlled environment (22-25 °C, 50% relative humidity, 12-h light-dark cycle). Mice aged 3 months were randomly assigned to one of the following four groups (n = 12 per group). (1) Control group: the mice were given a standard diet (30 ppm Zn^2+^) and deionized water *ad libitum*. (2) Zinc group: the mice were given a standard diet and deionized water containing zinc sulfate (ZnSO_4_, 20 mg/mL). (3) Zinc + CQ group: the mice were given a standard diet and deionized water containing ZnSO_4_ (20 mg/mL). At the age of 7 months, the mice were gavaged daily with CQ (30 mg/kg; Sigma-Aldrich, USA) dissolved in 0.05% carbo-xymethylcellulose (CMC; Sigma-Aldrich). (4) CQ group: the mice were given a standard diet and water, and gavaged daily with CQ (30 mg/kg) dissolved in 0.05% CMC starting from the age of 7 months. The doses of zinc and CQ used in this study were chosen based on previous reports that showed no serious toxicity in C57BL/6 mice after long-term treatment [16, 23, 34]. Additional APP/PS1 mice were given a standard diet and water until the age of 22 months (n = 4). The animal body weights were monitored, and their general health status was observed on a daily basis.

This study was carried out in accordance with the recommendations of “Laboratory Animals-Guideline of Welfare and Ethics, The Ethics Committee for Medical Laboratory Animals of China Medical University”. The protocol was approved by the Ethics Committee for Medical Laboratory Animals of China Medical University.

### Atomic absorption spectrum

Blood samples were collected from the retro-orbital plexus of 9-month-old mice of the indicated groups, under deep anesthesia with sodium pentobarbital (50 mg/kg, intraperitoneal administration [i.p.]). The mice were then euthanized by decapitation. The zinc levels in blood serum and brain were measured using a polarized Zeeman atomic absorption spectrophotometer (Hitachi 180-80, Japan).

### Tissue preparation

Mice were anesthetized with sodium pentobarbital (50 mg/kg, i.p., n = 5 *per* group) at the age of 9 months and euthanized by decapitation. The brains were removed immediately and split sagitally into halves. The left hemisphere was kept at -80 °C for Reverse-Transcription Polymerase Chain Reaction (RT-PCR) and Western blotting. The right hemisphere was immersed in 4% paraformaldehyde and sectioned with a cryostat at a thickness of 30 μm for confocal laser scanning microscopy (SP2; Leica, Germany). The 22-month-old mice were perfused with ice-cold saline. Their brains were dissected out and frozen in liquid nitrogen. Sections were cut with a cryostat at a thickness of 15 μm and placed on top of glass coverslips coated with 1× poly-L-lysine. The sections were stored at -20 °C until use.

### Cell culture and assessment of cell viability

COS-7 cells were incubated in Dulbecco's Modified Eagle Medium (DMEM; Thermo Fisher Scientific, USA) supplemented with 10% heat-inactivated fetal bovine serum (FBS; Thermo Fisher Scientific), 100 IU/ml penicillin, and 100 μg/ml streptomycin, at 37 °C in a 5% CO_2_ atmosphere. When the cells reached a confluence of nearly 70%, they were cultured in serum-free medium for 2 h. The cells were then incubated with ZnSO_4_ alone (0 µM, 50 µM, 100 µM, 150 µM, or 200 µM), or ZnSO_4_ (150 µM) with recombinant human S100A6 (hS100A6) protein (0 µg/mL, 100 µg/mL, 150 µg/mL, 200 µg/mL, or 300 µg/mL) addition for 12 h. The concentrations of zinc used in the cell viability experiments were selected based on a routine MTT [3-(4,5-dimethylthiazol-2-yl)-2,5-diphenyltetrazolium bromide] and lactate dehydrogenase (LDH) assays. The full-length recombinant hS100A6 protein in non-tagged version (Qiang Yao Technology, China) was dissolved in PBS.

COS-7 cells were transfected with pcDNA3.1-hS100A6 (provided by Prof. Anna Filipek, Laboratory of Calcium Binding Proteins, Nencki Institute of Experimental Biology of the Polish Academy of Science, Poland) or pcDNA3.1 vector as a control. All transfection experiments were performed according to the manufacturer’s protocol for Lipofectamine 2000 (Thermo Fisher Scientific). The cells were harvested at 24, 48, and 72 h post-transfection for RT-PCR or Western blotting. After 48-h incubation, the cells were treated with ZnSO_4_ (0 µM, 50 µM, 100 µM, 150 µM, and 200 µM) for 12 h.

Cell viability was assessed in 96-well plates by a quantitative colorimetric assay using MTT. Briefly, after treatment for 12 h, 500 μg/mL MTT was added to the medium, and the cells were incubated at 37 °C for 3 h. After removing the MTT solution, dimethyl sulfoxide was added to dissolve the colored formazan crystals. The absorbance of each well at 492 nm was measured using a Sunrise RC microplate reader (Tecan Group, Switzerland). Cell viability was expressed as the ratio of the measurements obtained from the treated and control samples. Lactate Dehydrogenase (LDH) Assay Kit (Abcam) was used to evaluate the presence of cells damage according to the manufacturer’s instructions. Measure output was performed at 450 nm on a microplate reader protected from light.

### Zinc-specific staining

The Zn^2+^-selective fluorophore 6-methoxy-(8-*p*-toluene-sulfonamido)-quinoline (TSQ; Thermo Fisher Scientific) has been widely used to detect intracellular zinc ions [35]. Briefly, fresh brain sections were incubated for 5 min with TSQ liquid. The samples were then washed with phosphate buffer (PB), and the zinc reaction products were examined under a fluorescence microscope (IX51, Olympus, Japan). To visualize zinc in cells, we used Zinquin ethyl ester (Dojindo Laboratories, Japan). The cells were cultured in 24-well plates after treatment with zinc-containing medium, or a mixture of zinc-containing medium and recombinant hS100A6 protein for 12 h. The cells were then incubated for 30 min in 0.24 µM Zinquin at room temperature, and the Zinquin-bound free zinc was examined using a fluorescence microscope.

### Co-incubation of S100A6 transfected cells and brain sections

Brain sections of 22-month-old APP/PS1 transgenic mice on coverslips were placed on COS-7 cells transfected with pcDNA3.1-hS100A6 or pcDNA3.1 (control). The sections and cells were co-incubated for 24 h at 37 °C in a 5% CO_2_ atmosphere [36]. Alternatively, the sections were cultured in DMEM with recombinant hS100A6 protein (300 μg/ml) or DMEM alone as a control for 24 h at 37 °C in a 5% CO_2_ atmosphere.

### Histochemistry, immunofluorescence and confocal laser scanning microscopy

Following co-incubation, brain sections from 22-month-old APP/PS1 transgenic mice were fixed in 4% paraformaldehyde for 1 h and washed with PB three times. The sections were then blocked with 5% bovine serum albumin for 1 h, and incubated with mouse anti-Aβ antibody (1:5,000; Sigma-Aldrich) overnight at 4 °C. After several rinses with PB, the sections were incubated with biotinylated goat anti-mouse IgG for 60 min at 37 °C. They were then rinsed in PB several times and treated with the ABC kit reagents for 1 h. After PB rinsing, the sections were incubated with a mixture of 0.025% 3,3-diaminobenzidine and 0.0033% hydrogen peroxide for 5 min. The sections were dehydrated and finally covered with neutral balsam. Images were collected and processed with the Adobe Photoshop software (Adobe Systems, USA). Control sections were incubated with normal serum instead of primary antibodies.

For immunostaining of S100A6 protein in the COS-7 cells transfected with pcDNA3.1-hS100A6, cells were fixed with 4% paraformaldehyde for 30 min. After several washes, the cells were blocked with normal donkey serum (NDS) (1: 20; Jackson Immuno Research Laboratory, USA) at room temperature for 30 min and then incubated with rabbit anti-S100A6 antibody (1:1,000; Novus, USA). After thoroughly rinsing, the cells were treated with a secondary antibody at room temperature for 2 h. The stainings were examined, and images were collected with an Olympus microscope.

To explore the localization of Aβ plaques and zinc ions, the brain sections were incubated with TSQ liquid for 5 min. After thoroughly rinsing, they were treated with 1% aqueous Thioflavin-S (Thio S) for 5 min. The TSQ binding and fibrillar Aβ plaques were imaged using a confocal laser scanning microscope. We also analyzed the alterations in the protein expression of Aβ and S100A6 with double immunofluorescence labeling. Briefly, brain sections were blocked with NDS (1:20; Jackson Immuno Research Laboratory) for 1 h and then incubated overnight in a mixture of two primary antibodies, mouse anti-Aβ (1:500) and rabbit anti-S100A6 (1:1,000; Novus). The sections were rinsed in PB several times and incubated for 2 h with a mixture of two secondary antibodies, FITC-conjugated donkey anti-mouse IgG (1:50) and Texas Red-conjugated donkey anti-rabbit IgG (1:50). After several rinses, the sections were sealed with anti-fading mounting medium and examined. Excitation filters for FITC (488 nm) and Texas Red (568 nm) were used for visualization. Images were obtained and processed using the Adobe Photoshop software. Several sections were incubated with NDS instead of primary antibodies as negative controls in every experiment.

**Figure 1.**
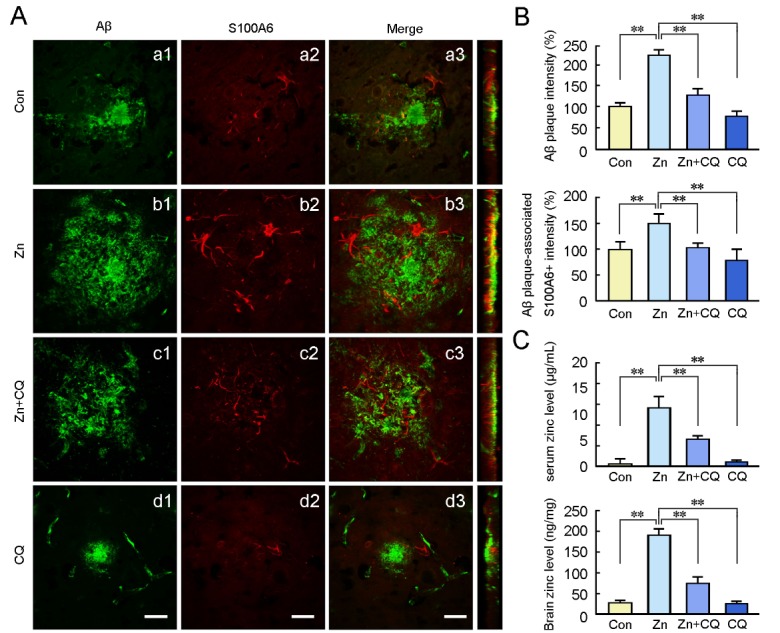
Confocal images showing the effects of zinc and chelator treatments on S100A6 expression and Aβ aggregation in the APP/PS1 mouse brain The 9-month-old APP/PS1 mice on high-zinc (Zn), zinc + clioquinol (Zn + CQ), or CQ diet were sacrificed. Age-matched APP/PS1 mice given a standard diet and deionized water were used as the controls (Con). (**A**) Frozen sections of the brain double immunostained with anti-Aβ (green) and anti-S100A6 (red) antibodies showing the distribution and expression of Aβ (a1-d1) and S100A6 (a2-d2), and their co-localization (a3-d3). Aβ and S100A6 immunostaining show significant co-localization in the brain sections of APP/PS1 mice on a high-zinc diet. The Y-Z images depicted in the right panel indicate the positive immunofluorescence staining after orthogonal sectioning. (**B**) The intensity of Aβ plaques and Aβ plaques-associated S100A6 immunofluorescence were determined. (**C**) Atomic absorption spectrum assay was used for the measurement of zinc levels in the cortex. Values are means ± S.E.M. Results were compared by a two-way ANOVA followed by *t* test (*n* = 5). **P < 0.01. Scale bars = 20 μm.

### Western blotting

Western blotting was performed as described previously [37]. Briefly, brain tissue fragments and cell pellets were homogenized in ice-cold lysis buffer overnight at 4 °C. The lysates were centrifuged at 12,000 rpm for 30 min at 4 °C. The supernatants were collected, and total protein was quantified using a bicinchoninic acid (BCA) protein assay kit (Pierce, USA). Proteins (60 µg) from each sample were separated on 8-15% sodium dodecyl sulfate (SDS) polyacrylamide gels and transferred onto difluoride (PVDF) membranes (Millipore, USA). The membranes were blocked in 5% fat-free milk for 1 h and incubated with anti-S100A6 (1:1,000) and anti-glyceraldehyde 3-phosphate dehydrogenase (GAPDH; 1:10,000; KC-5G5; Kang Chen, China) primary antibodies at 4 °C, overnight. The membranes were then washed and incubated with a horseradish peroxidase (HRP)-conjugated secondary antibody (1:5,000; Santa Cruz Biotechnology, USA) for 2 h at room temperature. The bands were visualized by incubation with Super Signal West Pico Chemiluminescent Substrate (Pierce). Band intensities were quantified using the Image-pro Plus 6.0 analysis software (Media Cybernetics, USA).

### Sandwich ELISA

Brain sections from mice that had received indicated treatment were collected and treated with 5 M guanidine HCl/50 mM Tris-HCl (pH 8.0) for protein dissolving. The levels of Aβ42 were analyzed with the human Aβ42 Ultrasensitive ELISA Kit (Thermo Fischer Scientific) according to the manufacturer’s instructions. The absorbance was read at 450 nm.

### RT-PCR

Total RNAs from homogenized cells and brain tissue samples were isolated using the TRIzol reagent (Thermo Fisher Scientific). RNA concentrations were determined by measuring absorbance at 260 nm. Total RNA (5 μg) from each sample was transcribed into cDNA using a reverse-transcription system kit (Promega, USA). Mouse S100A6 (mS100A6) and hS100A6 cDNAs corresponding to GenBank sequences were amplified using the following primers: mS100A6: forward: 5’-CCTTC TCGTGGCCATCTT-3; and reverse: 5’-CCCAGGAA GGCGACATAC-3; hS100A6: forward: 5’-TCAGCCA TGGCATGCCCCCTGGAT-3’; and reverse: 5’-ATATT TTTCAGCCCTGAGGGCTTC-3’. The PCR parameters were as follows: initial denaturation at 95 °C for 5 min, 30 amplification cycles (95 °C for 40 s, 58 °C for 40 s, and 72 °C for 40 s), and final extension at 72 °C for 10 min. The control mouse and human GAPDH cDNA sequences were amplified using the following primers: mouse GAPDH: forward: 5’-TGGCAAAGTGGAGA TTGTTG-3’; and reverse: 5’-GTCTTCTGGGTGGCA GTGAT-3’; human GAPDH: forward: 5’-GGATTTGGTCGTATTG GG-3’; and reverse: 5’-TCGCTCCTG GAAGATGG-3’. The PCR products were electrophoresed simultaneously on a 2% ethidium bromide-stained agarose gel. The results were confirmed and quantified with the ChemDoc XRS Quantity One software (BioRad, USA).

### Statistical analysis

The results are expressed as the mean ± standard error of the mean (S.E.M.). All statistical analyses were performed using the SPSS 18.0 software. One-way or two-way analysis of variance (ANOVA) were performed to compare two means. Differences with *p* < 0.05 were defined as statistically significant.

## RESULTS

### Effects of zinc or CQ treatments on S100A6 expression and Aβ aggregation in the APP/PS1 mouse brain

We first examined whether the chronic intake of high dietary zinc or treatment with the chelator, CQ, could affect Aβ deposition and S100A6 expression. Double immunofluorescence staining for Aβ and S100A6 was performed to analyze colocalization of Aβ and S100A6 protein in the brains of APP/PS1 mice. Aβ-positive staining showed typical characteristics of senile plaques (Fig. 1A, a1-d1). S100A6 was expressed prominently in the peripheral part of the plaques (Fig. 1A, a2-d2). The intensity of the Aβ-immunoreactive senile plaques (Fig. 1A, b1) and S100A6 immunostaining (Fig. 1A, b2) were markedly increased in the brains of zinc-treated mice compared with those of control mice (Fig. 1A, a1, a2) (*P* < 0.01, Fig. 1B). After treatment with CQ (Zn + CQ), the intensity of the senile plaques was smaller than that in the zinc group. S100A6 expression was also decreased in the Zn + CQ group (Fig. 1A, c2). In addition, the intensity of Aβ-immunoreactive senile plaques was reduced (*P* < 0.01, Fig. 1A, d1) and S100A6 staining nearly disappeared in the CQ treatment group (Fig. 1A, d2). Further, the distribution of S100A6 did not overlap substantially with Aβ immunofluorescence in any group (Fig. 1A, a3-d3). Zinc levels in blood serum and brain were determined by atomic absorption spectrum. As shown in Fig. 1C, the high zinc diet caused a significant increase in serum zinc levels of APP/PS1 mice relative to control group (*P* < 0.01), and brain zinc levels were increased, as well (*P* < 0.01). The variances in the serum zinc levels in the Zn + CQ and CQ group were not statistically significant when compared with the control group (*P* > 0.05). CQ treatment did not lead to a marked difference in the zinc levels of the APP/PS1 mouse brain relative to controls although there was a trend towards decrease (*P* > 0.05).

**Figure 2.**
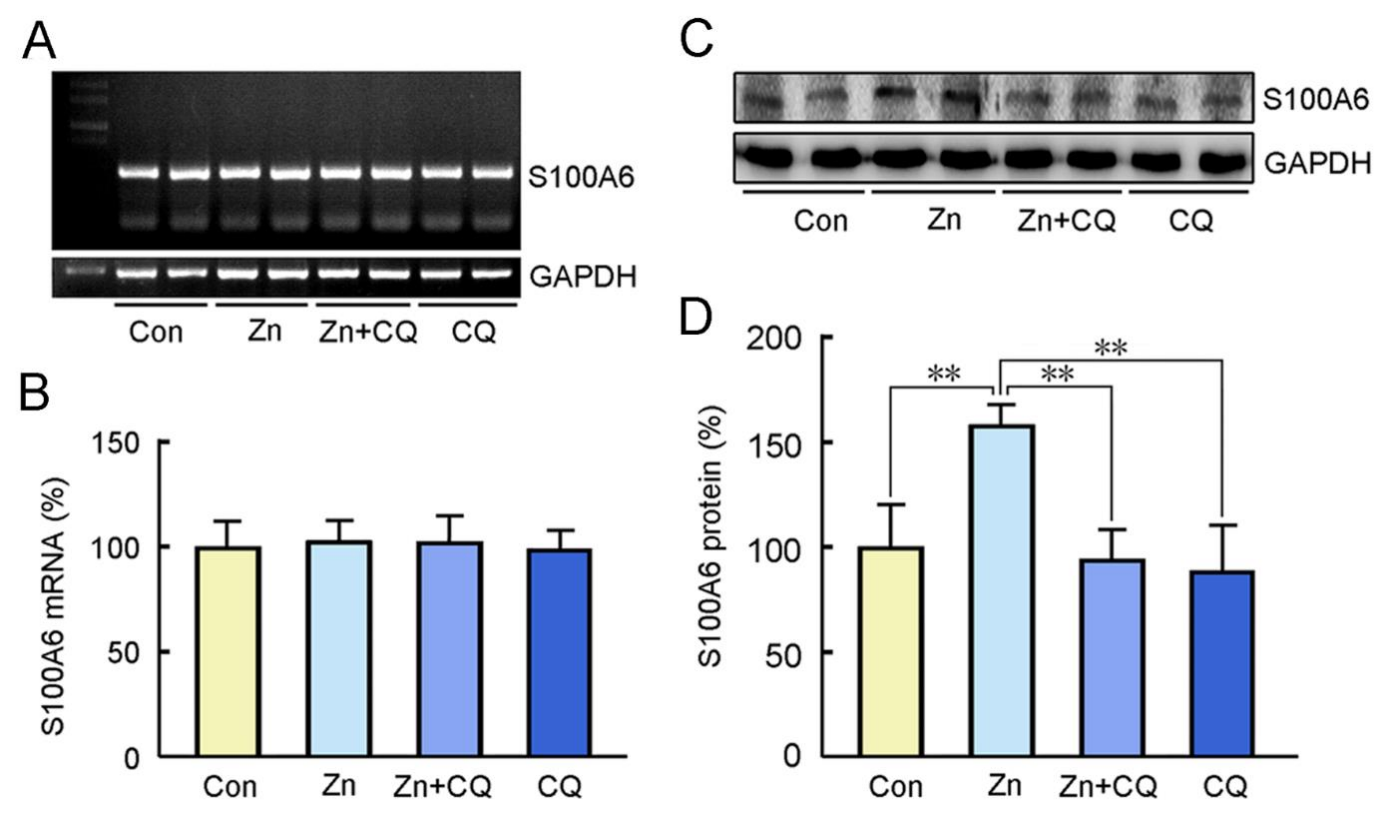
High-zinc diet led to an increase of S100A6 protein expression in the brains of APP/PS1 mice RT-PCR (A, B) and Western blot (C, D) assay were used to detect the mRNA and protein levels of S100A6, respectively, in the brains of APP/PS1 mice fed with high-zinc, zinc + CQ, or CQ diet. Age-matched APP/PS1 mice administered with standard diet and deionized water served as controls (Con). The results are presented as percentages, and the control is defined as 100%. Values represent means ± S.E.M. Results were compared by a two-way ANOVA followed by *t* test, **P < 0.01 versus the controls, ## *P* < 0.01 versus the zinc treatment group (*n* = 5).

### S100A6 protein levels increased in the brains of APP/PS1 mice fed a high-zinc diet

We tested whether a high dose of dietary zinc affected S100A6 expression. The mRNA and protein levels of S100A6 were measured by RT-PCR and Western blotting, respectively. The expression levels of S100A6 mRNA in the brain were not significantly different among APP/PS1 mice treated with zinc sulfate, CQ, or their combination, and the control group (Fig. 2A, B). Western blot analysis revealed that zinc treatment significantly increased S100A6 protein level compared with that in controls fed a normal diet (Fig. 2C, D). The S100A6 protein levels were significantly reduced in the CQ and zinc sulfate + CQ mice compared with those in mice fed a high-zinc diet.

### S100A6 protein treatment decreased zinc-induced cell toxicity

**Figure 3.**
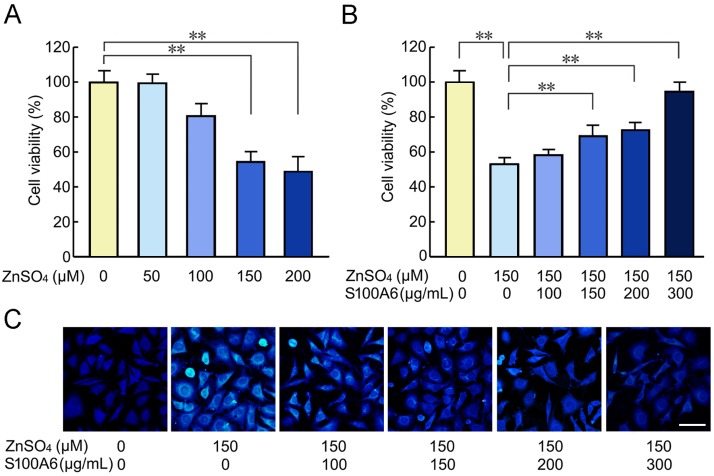
Recombinant human S100A6 (hS100A6) protein reversed zinc-induced cell toxicity The MTT assay results show the changes in the cell viability with the addition of indicated concentrations of zinc sulfate (ZnSO_4_) (A) and different concentrations of hS100A6 protein along with 150 μM of ZnSO_4_ (B). Cells treated with the vehicle served as controls. Zinquin staining was used to detect zinc in the cells that were incubated with 150 μM ZnSO_4_ and different concentrations of hS100A6 protein (C). Values are means ± S.E.M. and are representative of at least three independent experiments. Results were compared by one-way ANOVA with *post-hoc* Fisher’s protected least significant difference (PLSD) test, **P < 0.01. Scale bars = 30 μm.

To avoid the influence of cell death in zinc toxicity experiments, we performed a cell viability assay to select an optimal ion concentration. Based on the results of this assay, 150 μM of zinc sulfate was chosen (Fig. 3A).

We tested whether recombinant hS100A6 protein could affect the viability of COS-7 cells treated with zinc ions. MTT assay results showed that cells treated with zinc sulfate (150 μM) and recombinant hS100A6 protein (150 μg/mL, 200 μg/mL, or 300 μg/mL) exhibited increased cell viability compared with cells cultured with zinc sulfate alone (Fig. 3B). The zinc-specific fluorescent probe Zinquin was used to examine the levels of zinc ions in COS-7 cells after treatment with zinc sulfate alone or in combination with recombinant hS100A6 protein. Intense Zinquin fluorescence was distributed in a punctate pattern around the nucleus (Fig. 3C). After high-zinc treatment (150 μM), the living cells appeared abnormal, and the cell processes disappeared. However, after co-incubation with zinc sulfate and recombinant hS100A6 protein, intracellular Zinquin staining was reduced in an S100A6-concentration-dependent manner, completely disappearing at 300 μg/mL (Fig. 3C).

**Figure 4.**
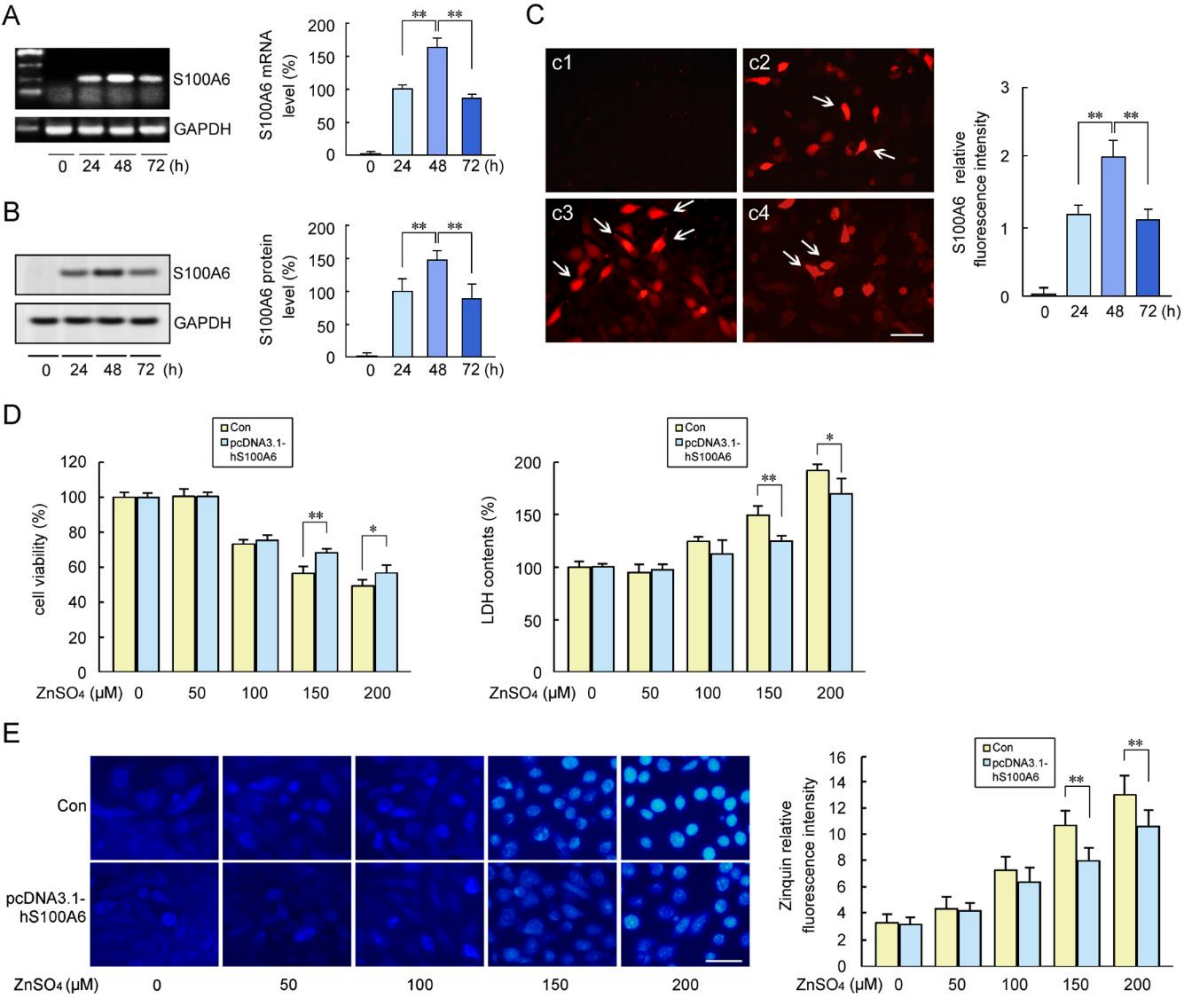
Overexpression of hS100A6 mitigated zinc-induced decrease of COS-7 cell viability COS-7 cells were transfected with pcDNA3.1-hS100A6 before being incubated with zinc sulfate (0 µM, 50 µM, 100 µM, 150 µM and 200 µM) for 12 h. COS-7 cells transfected with pcDNA3.1 vector were used as controls (Con). (**A**) The S100A6 mRNA levels were evaluated using RT-PCR at 48 and 72 h after transfection. (**B**) Western blot analyses demonstrated S100A6 protein overexpression in the transfected COS-7 cells and in controls. The levels of S100A6 mRNA and protein were expressed as the ratio of the mean intensity at indicated time to the level at 24 h after transfection. (**C**) Immunofluorescence staining for S100A6 indicate the representative images S100A6-positive cells (Arrows) confirmed the successful transfection of COS-7 with pcDNA3.1-hS100A6 (c1: 0 h; c2: 24 h; c3: 48h; c4: 72h). (**D**) MTT and lactate dehydrogenase (LDH) assays were performed to determine cell viability after addition of the indicated concentrations of zinc sulfate. The control was defined as 100%. (**E**) Intracellular zinc levels were detected using the Zinquin staining. Values are means ± S.E.M. and are representative of at least three independent experiments. Results were compared by one-way ANOVA with *post-hoc* Fisher’s protected least significant difference (PLSD) test, *P < 0.05; **P < 0.01. Scale bars = 30 μm.

Next, we examined the effect of hS100A6 overexpression in COS-7 cells on their viability upon incubation with zinc ions. First, we tested whether hS100A6 overexpression affected the endogenous S100A6 protein levels. The cells were harvested at 24, 48, or 72 h after transfection with pcDNA3.1-hS100A6 and subjected to RT-PCR and Western blot analyses. Both RT-PCR and immunoblotting revealed significant increases in endogenous S100A6 levels following hS100A6 overexpression at 48 and 72 h (Fig. 4A, B), and immunofluorescence labeling of S100A6 showed a specific immunostaining in the hS100A6-transfected COS-7 cells, especially at 48 h after transfection (Fig. 4C). Second, we examined the effect of hS100A6 transfection on cell viability. Forty-eight hours after transfection, the cells were treated with zinc sulfate at different concentrations. MTT and lactate dehydrogenase (LDH) assays revealed that cell viability was dramatically increased in COS-7 cells overexpressing hS100A6 compared with that of the control cells treated with 150 μM or 200 μM zinc sulfate (Fig. 4D). Zinquin staining showed that zinc fluorescence was present around the nuclei, and the intensity of zinc fluorescence increased with increasing concentrations of zinc sulfate in the 0-200 μM range. In contrast, after transfection with pcDNA3.1-hS100A6, zinc fluorescence intensity was decreased compared with the control group (Fig. 4E).

### Exogenous S100A6 treatment reduced zinc levels and senile plaques in the aged APP/PS1 mouse brain sections

**Figure 5.**
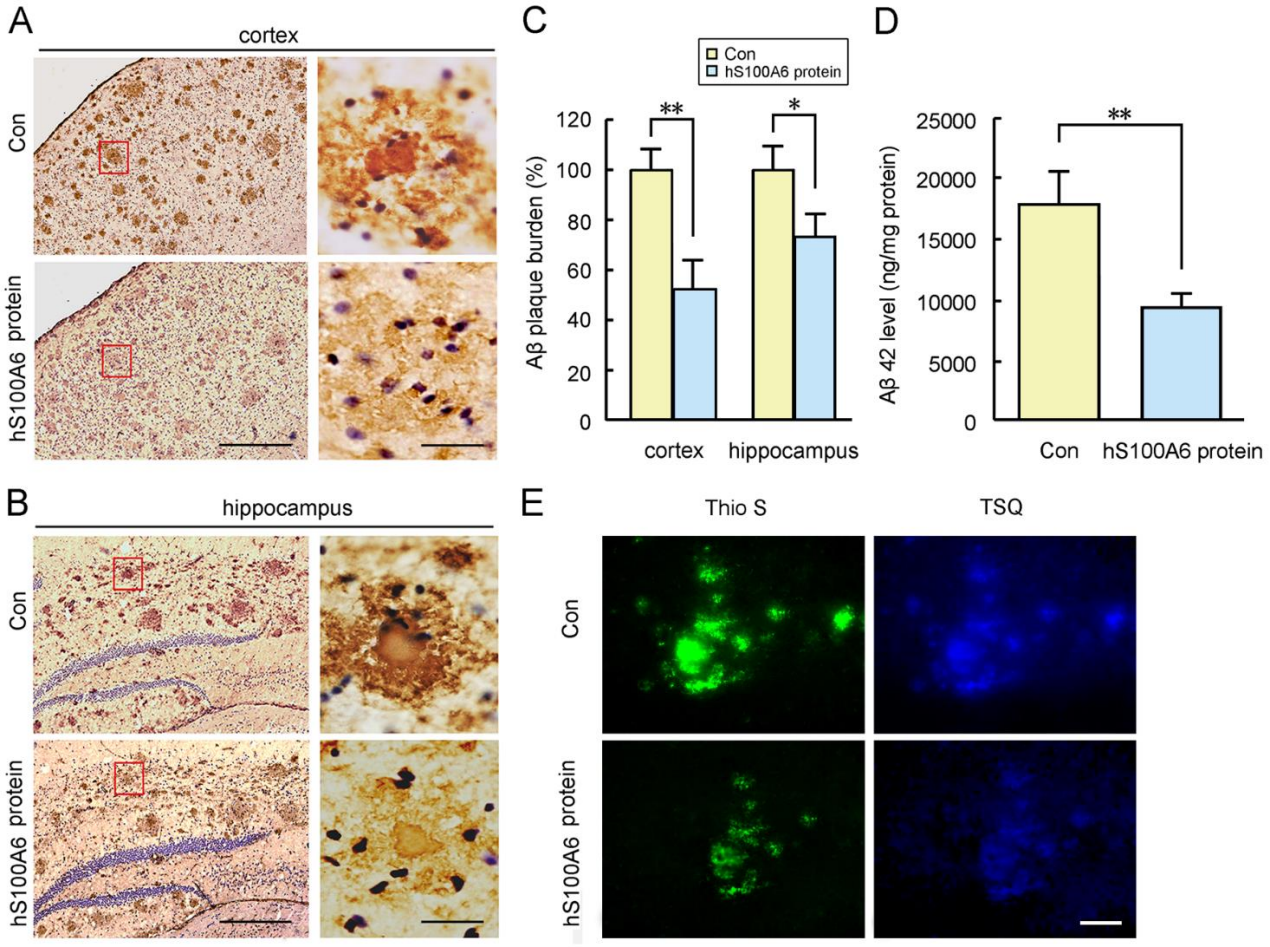
Recombinant hS100A6 protein contributed to Aβ degradation in brain slices of the APP/PS1 mouse Brain slices collected from APP/PS1 mouse were incubated in Dulbecco’s Modified Eagle Medium (DMEM) supplemented with recombinant hS100A6 protein for 24 h. Adjacent sections from the same brains incubated in DMEM medium alone were used as controls (Con). Immunohistochemical staining with anti-Aβ antibody demonstrated Aβ protein expression. Representative images indicating the Aβ deposits in the cerebral cortex (A) and hippocampus (B). High-magnification images of representative Aβ-positive staining are shown in the right panels. (**C**) The Aβ plaque burden were quantified. (**D**) Aβ42 levels were determined by ELISA assay. The content of Aβ42 was expressed as ng per mg of tissue protein. (**E**) Thioflavin-S (Thio S) and N-(6-methoxy-8-quinolyl)-p-toluenesulfonamide (TSQ) stainings showed the distribution of fibrillar Aβ and zinc, respectively, in the slices of APP/PS1 mouse brain incubated with hS100A6 protein or medium alone. Values are means ± S.E.M. Results were compared by Student’s *t* test (n = 4). *P < 0.05, **P < 0.01 versus the controls. Scale bars: A, B = 200 μm, and 20 μm in the high magnification of right panels; D = 20 μm.

**Figure 6.**
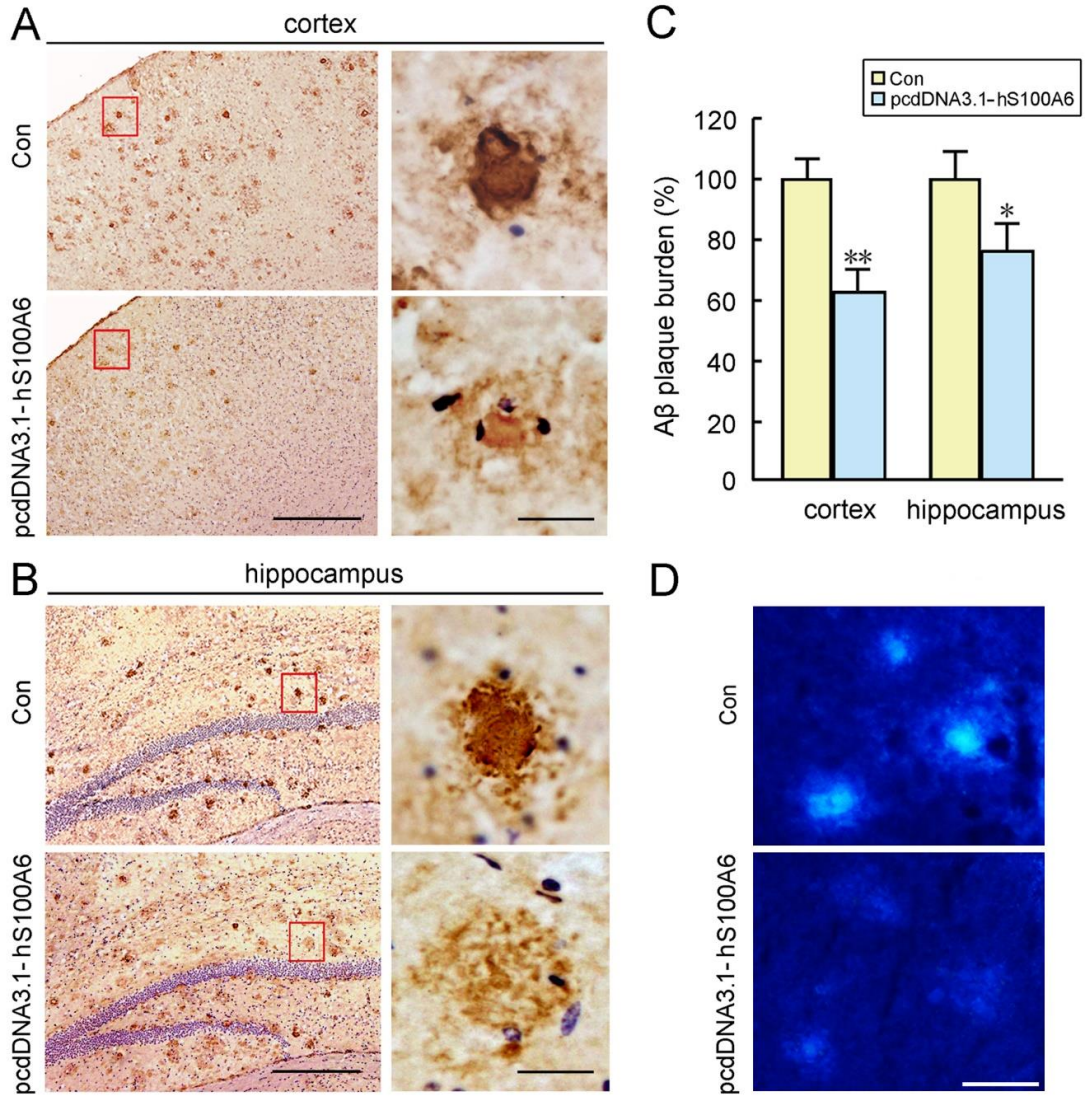
Overexpression of hS100A6 was associated with the clearance of amyloid plaques in brain sections of the APP/PS1 mice Brain slices and adjacent sections from the same brains collected from APP/PS1 mouse were co-incubated with COS-7 cells transfected with pcDNA3.1-hS100A6 or with pcDNA3.1-hS100A6 empty vector (control, Con), respectively, for 24 h. Representative immunohistochemistry images showing the Aβ plaques in the cerebral cortex (A) and hippocampus (B) of the APP/PS1 mice brain. The right panels depict representative Aβ plaques in high magnification. Quantification of Aβ burden demonstrated a significant decrease in the sections incubated with COS-7 cells expressing hS100A6 compared to controls (C). (**D**) TSQ fluorescence staining was performed to detect zinc levels in the slices of APP/PS1 mouse brain co-incubated with cells expressing hS100A6 or controls. Values are means ± S.E.M. Results were compared by Student’s *t* test (n = 4). *P < 0.05, **P < 0.01 versus the controls. Scale bars: A, B = 200 μm, and 20 μm in the high magnification right panels; D = 20 μm.

To study the effects of S100A6 on Aβ deposits present in APP/PS1 mice, APP/PS1 mouse brain sections were co-incubated with recombinant hS100A6 protein or COS-7 cells transfected with pcDNA3.1-hS100A6 for 24 h, followed by immunohistochemical Aβ detection. We used adjacent sections from the same brains for a valid comparison. As shown in Figure 5, the Aβ-immuno-reactive deposits appeared intact in the control sections, with a dense core of tightly aggregated amyloid fibrils surrounded by more radially oriented fibrils in the cortex (Fig. 5A) and hippocampus (Fig. 5B). The Aβ burden of brain sections treated with recombinant hS100A6 protein was significantly reduced compared to those in adjacent sections incubated with medium alone (control) (Fig. 5C). Determination of human Aβ42 levels using ELISA analysis confirmed hS100A6-triggered decreases of Aβ42 contents (*P* < 0.01, Fig. 5D). The zinc levels in the Aβ plaques of APP/PS1 mouse brain sections were detected with the zinc-specific fluorescent probe, TSQ. As shown in Figure 5E, typical TSQ fluorescence was observed in the Thioflavin-S (Thio S) plaques of APP/PS1 mouse brain, whereas TSQ staining was faint in the sections of hS100A6 protein treatment group. Moreover, we co-incubated sections of APP/PS1 mouse brains with COS-7 cells transfected with pcDNA3.1-hS100A6. Adjacent sections co-incubated with COS-7 cells transfected with pcDNA3.1-vector were used as controls (Con). As shown in Figure 6, the levels of Aβ immunostaining in the cortex (Fig. 6A) and hippocampus (Fig. 6B) in sections incubated with S100A6 transfected COS-7 cells were reduced compared to those in the matching areas of controls, and the remaining Aβ deposits had a low-density core surrounded by loose amyloid fibrils. TSQ fluorescence density was less in the sections co-incubated with COS-7 cells overexpressing hS100A6 relative to adjacent sections co-incubated with control COS-7 cells (Fig. 6D).

## DISCUSSION

The central event of AD pathology is the cerebral accumulation and targeted deposition of Aβ. Mounting evidence suggests that both APP and its proteolytic product Aβ are involved in zinc homeostasis in the AD brain [38, 39]. Zinc ions are enriched in the neocortex, and significantly concentrated in the Aβ plaques in AD [40]. Aβ1-16 can coordinate up to three zinc ions, binding to histidines 6, 13 and 14 [41]. The binding of zinc to Aβ rapidly induces Aβ aggregation [42]. In the brains of APP/PS1 transgenic mice, zinc plays an important role not only in the formation of senile plaques, but also in neuronal toxicity and damage through activating multiple intracellular pathways [43, 44]. Brain metal-binding protein has been shown to be the underlying regulator of metal homeostasis in the AD brain [45, 46]. Aβ clearance might be promoted through specific modulation of metal-induced effects or modification of metal-protein interactions [15]. The mechanism by which Aβ levels are reduced is believed to involve amyloid plaque solubilization by the removal of Aβ-bound zinc ions [24].

S100A6 is a Ca^2+^/Zn^2+^-binding protein [25, 26], having two Zn^2+^-binding sites. One involves cysteine, whereas, the other remains uncharacterized [30]. The affinity of S100A6 for Zn^2+^ is up to 300 times stronger than its ability to bind Ca^2+^ [30]. S100A6 has been shown to be upregulated in AD patients and the distributed patterns of fibril Aβ and astrocytic S100A6 immunoreactive areas are similar in cases of sporadic AD [29]. We proposed that S100A6 upregulation might suggest its involvement in the regulation of zinc homeostasis in the AD brain. In the present study, we first evaluated the correlation between S100A6 expression and zinc levels in the APP/PS1 mouse brain. Our results showed that brain zinc overload *via* chronic administration of a high-zinc diet enhanced Aβ aggregation in the brains of APP/PS1 transgenic mice, which was consistent with our previous findings [16]. In addition, the expression of S100A6 around senile plaques was increased along with the increases of Aβ deposition. However, S100A6 and Aβ expression were substantially reduced in the brains of transgenic mice fed with zinc and CQ, and nearly disappeared in those fed with CQ alone. Thus, the expression of S100A6 around the plaques and increased Aβ expression were associated with the presence of a high concentration of zinc in the brain. Total zinc levels in the APP/PS1 mouse brain were not significantly altered under CQ treatment, suggesting CQ-triggered improvement of zinc homeostasis rather than strict zinc chelating. In the present study, zinc or CQ treatment did not alter the mRNA levels of S100A6. The increase of S100A6 in the brain of zinc-administered APP/PS1 mouse only occurred at the protein level. Further mechanistic studies may allow us to discover the regulatory pathways.

It has been reported that conditional overexpression of S100A6 in cardiac myocytes can mitigate hypertrophy and apoptosis of myocytes after myocardial infarction [47]. Mofid and colleagues [48] reported that S100A6 overexpression attenuated myocardial ischemia reperfusion injury. Fang and colleagues [49] showed that the recovery of the cognitive function of rats with traumatic brain injury was related to the re-elevation of S100A6 mRNA levels in the hippocampus. Considering the ability of S100A6 to bind zinc, we proposed that the upregulation of S100A6 in AD patients and APP/PS1 transgenic mouse brains might play a buffering role and protect against high zinc levels. Double immunofluorescence staining for S100A6 and Aβ showed that the expression of S100A6 occurred around the senile plaques, and S100A6 and Aβ did not colocalize. This result indicated that S100A6 may be involved in Aβ clearance in the APP/PS1 mouse brain. S100A6 expression at the protein or mRNA level can be upregulated by multiple factors [50-54]. Although we found S100A6 expression to be activated by zinc, the mechanisms of this upregulation are unclear. In the present study, to investigate whether S100A6 could compete with Aβ for zinc, mitigating Aβ-zinc aggregation, we explored the effects of S100A6 on zinc levels in COS-7 cells *in vitro*. S100A6 is not expressed in the COS-7 cell line [55], eliminating the potential interference of endogenous S100A6 [55]. Considering that zinc ions beyond a narrow concentration range are harmful to several cell types *in vitro* [56], we performed a zinc ion concentration screening. Zinc-selective fluorophore Zinquin was used to detect intracellular zinc ions. COS-7 cells cultured with high zinc concentrations showed reduced zinc staining after treatment with recombinant hS100A6 protein or transfection with a hS100A6 expression vector. In addition, both recombinant and overexpressed hS100A6 increased the number of surviving COS-7 cells after exposure to high Zn^2+^ treatment. These results suggest that S100A6 can reduce zinc toxicity in cultured cells through binding free zinc ions.

To further characterize the possible role of S100A6 upregulation in the AD brain, we co-incubated COS-7 cells overexpressing hS100A6 with, or added recombinant hS100A6 protein to, unfixed Aβ-rich brain sections from aged APP/PS1 transgenic mice. In addition, we used another zinc-selective fluorophore, TSQ, to detect Aβ-associated zinc in APP/PS1 brain sections. Interestingly, it appeared that the intensity of TSQ staining of zinc was reduced in the hippocampus and cortex in sections incubated with recombinant hS100A6 protein or COS-7 cells transfected with hS100A6. Confocal laser scanning microscopy assays with Thio S and TSQ showed that hS100A6 treatment-induced decrease of fibrillar Aβ plaques was accompanied by a decline of zinc levels. In addition, Aβ immunohistochemical analysis of sections treated with recombinant hS100A6 protein or co-incubated with hS100A6-overexpressing COS-7 cells revealed a disrupted Aβ plaque morphology, with the remaining Aβ deposits showing a low-density core surrounded by loose amyloid fibrils. The evaluation of Aβ42 levels by ELISA confirmed these results, suggesting that S100A6 may lead to Aβ depolymerization by sequestering zinc. A limitation of our study was that S100A6 immunostaining in the APP/PS1 mouse brain could not provide direct evidence of S100A6 sequestering zinc* in vivo*. There are several uncertainties remaining on the combining pattern of S100A6 and zinc in the AD patient’s brain. Further animal experiments with conditional, neural specific, S100A6 transgenic or S100A6 knockout mice are necessary to delineate the effect of S100A6 on zinc modulation.

To the best of our knowledge, the present study is the first to report a correlation between zinc levels and S100A6 expression. Chronic exposure to a high-zinc diet led to increases in S100A6 expression and Aβ deposition in the brains of APP/PS1 transgenic mice. Moreover, exogenous S100A6 induced Aβ disaggregation in APP/PS1 mouse brain sections through zinc binding. Thus, S100A6 upregulation in the AD brain may be a protective mechanism to sequester zinc and reduce amyloid deposition. S100A6 may therefore be a potential target for AD prevention and therapy. Further studies of S100A6 function in AD animal models and patients with AD are essential.
